# Brief Repeated Attention Training for Psychological Distress: Findings from Two Experiments

**DOI:** 10.3390/bs15081052

**Published:** 2025-08-03

**Authors:** David Skvarc, Shannon Hyder, Laetitia Leary, Shahni Watts, Marcus Seecamp, Lewis Burns, Alexa Hayley

**Affiliations:** School of Psychology, Deakin University, Geelong, VIC 3216, Australia

**Keywords:** attention, mood dysfunction, psychological distress, remote interventions, bias modification

## Abstract

Psychological distress is understood to be maintained by attention. We performed two experiments examining the impact of attention training (AT) on psychological distress symptoms. *Experiment one* (*N* = 336) investigated what effects might be detected in a simple experimental design with longitudinal measurements, while *experiment two* (*N* = 214) examined whether using a different emotional stimulus could induce an immediate anxiolytic effect in response to AT. Attentional biases were operationalized as the target search latency correlated with mood and psychological distress scores. While limited evidence of attentional biases was found in participants with higher mood distress, correlations emerged in the experimental conditions at day thirty, indicating a relationship between task latency, stress, and changes in depression (experimental one). We found no immediate between–within-group differences in outcome when including different emotional stimuli (experiment two). Despite attentional biases being less apparent in community samples, attentional training for bias modification was effective in eliciting positive biases, leading to improved mood. Notably, participants in the control condition reported the greatest mood and psychological distress improvements, whereas changes in the experimental condition primarily pertained to attentional biases. Taken together, these findings suggest that AT tasks can improve distress, but not through changes in attentional biases.

## 1. Introduction

Many psychological distress states are associated with attentional and cognitive biases, which refer to biased thought processes that are purported to occur near-automatically (Matheson et al., 2019). Distressed individuals display attentional biases (AB) through preferentially attending to emotion-congruent information. Our understanding of AB has primarily stemmed from research examining anxious populations, where individuals display increased engagement with threatening information (i.e., threat-related AB; Bar-Haim et al. (2007)). Anxious individuals show a compromised threat discrimination system, whereby stimuli with lower levels of emotional valence are interpreted as having a disproportionately high threat value, triggering an over-generalized fear response (Dennis-Tiwary et al., 2019). As such, anxious attentional systems are sensitive to, and biased in favor of, threat-related information in their environment (Bar-Haim et al., 2007).

Threat Avoidance Training is one form of attentional training (AT) operating on the fundamental assumption that anxious individuals display enhanced vigilance toward threats and proposes that anxiety symptoms should improve by reducing the magnitude of AB (Mogg et al., 2017; Mogoaşe et al., 2014). Though initial results were promising and demonstrated significant changes to both AB and anxiety symptoms (Hakamata et al., 2010), subsequent studies have failed to replicate these findings, with inconsistent observations of AB and anxiolytic effects (Cristea et al., 2016; Mogoaşe et al., 2014). Mogg et al. (2017) suggested that reductions in anxiety were primarily restricted to lab-based experiments that occurred across both experimental and control conditions, and that reducing pre-existing AB toward threat does not necessarily produce anxiolytic effects. As such, other cognitive mechanisms, such as attentional control, are likely to contribute to therapeutic improvement, encouraging a more comprehensive exploration of alternative AT methods, including Positive Search Training (PST).

PST explicitly encourages individuals to search a visual matrix for a single positive stimulus amongst an array of threat cues (Mogg & Bradley, 2016). In a series of experiments, Dandeneau et al. (2007) demonstrated the unique potential of PST using the “face-in-the-crowd” visual search (VS) paradigm, where participants repeatedly identified a single smiling face amongst angry, threatening faces. The results proved highly efficacious, with participants indicating lower physiological and self-reported stress levels. Following this, several studies have utilized the “face-in-the-crowd” positive search paradigm with similar success. Specifically, Waters et al. (2013) found that more than half of their clinical pediatric sample no longer met the criteria for their principal anxiety diagnosis following PST, and improvements were maintained after six months.

Unlike Threat Avoidance Training, PST does not rely on the assumption that anxiety is characterized by enhanced vigilance towards threat and instead focuses on promoting adaptive functioning by engaging in controlled, top-down cognitive processes (Mogg et al., 2017; Waters et al., 2013). By explicitly providing individuals with the goal of identifying positive stimuli, it is possible that PST increases inhibitory control and prioritizes finite attentional resources to purposefully orient towards stimuli associated with safety. Doing so may allow individuals to override the bottom-up signaling of potential threats presented through the angry faces in the array. By increasing attentional control, anxious individuals may have a greater capacity to reappraise threatening stimuli as goal-irrelevant, thereby violating their danger expectancies (Waters et al., 2013). Replacing maladaptive coping strategies with skillful emotion regulation techniques likely transfers to future stressors and contributes to the maintained anxiolytic effect observed in initial PST trials (Waters et al., 2013).

However, not all studies utilizing the “face-in-the-crowd” paradigm have yielded such encouraging results. For example, De Voogd et al. (2017) observed a large reduction in AB but no associated effects on anxiety symptoms following training. However, given that only a handful of studies with relatively small sample sizes have examined the “face-in-the-crowd” VS paradigm, it is important to interpret these initial results cautiously. Moreover, it is unclear if the anxiolytic effects of PST depend on the use of highly emotive visual arrays (e.g., positive smiling stimuli among negative threatening stimuli) and whether arrays with lower thresholds of emotional valence might produce similar effects (Mogg & Bradley, 2016). As such, the AT literature must continue exploring different iterations of this novel paradigm to clarify whether this is an effective intervention tool for anxious populations.

The impact of state anxiety, immediate anxiety symptoms occurring in response to distress, is one area that the AT literature has often overlooked. State anxiety is a transitory condition of heightened arousal that is often evoked through unpredictable, stressful situations and can lead to clinical anxiety (Bishop et al., 2004). The cognitive literature identified that individuals presenting with state anxiety display threat-related AB equivalent to clinically anxious individuals, suggesting that a clinical cut-off is of little significance when examining biased attentional processes (Basanovic et al., 2021; Bourke et al., 2010; Nelson et al., 2015). However, much of the AT literature has utilized clinical samples, likely based on the assumption that clinical samples present more robust threat-related bias (Bar-Haim et al., 2007). Of the few studies that have examined the influence of AT on state anxiety, the evidence indicates significant symptom reductions (Brosan et al., 2011; See et al., 2009). However, most of these studies utilized Threat Avoidance Training, which is potentially less efficacious. Two studies have explored the effect of PST on state anxiety, with varying levels of success. While Dandeneau et al. (2007) observed significant improvements in state anxiety, this improvement was limited to the context of exam-related anxiety and did not translate to other stressors. Further, while De Voogd et al. (2017) intended to induce state anxiety before participants completing the VS paradigm experimentally, their attempts remained unsuccessful, with participants reporting no indication of state anxiety. As such, little is known about whether AT effectively attenuates state anxiety symptoms in response to psychological distress, and whether newer modalities, such as the “face-in-the-crowd” VS paradigm, can induce more pronounced results.

### The Current Study

The current study consists of two experiments that examine the efficacy of the AT “face-in-the-crowd” VS paradigm as a brief intervention tool to improve psychological distress symptoms. The first experiment examines a simple active versus control AT intervention and sought to investigate the presence or absence of an AB, as well as the efficacy of the intervention for psychological distress. The second experiment examines a single intervention consisting of four AT conditions with varying intensities of valence to understand their differential effect on improving state psychological distress measured immediately before and after the AT.

## 2. Materials and Methods

Both experiments were online, randomized, between–within mixed-design experiments using Qualtrics and Inquisit 5 software. Participants were required to be at least 18 years old, but no other eligibility requirements were used. For both experiments, a combination of social media advertising and snowballing was used; we placed advertisements through Facebook, Instagram, SurveyCircle, and SurveySwap. The recruitment phase for experiment one ran from March 2020 to August 2021, and the recruitment phase for experiment two ran from March to August 2022. Participants were offered the opportunity to enter themselves into a prize draw for an AUD 50 gift voucher as an incentive.

For each experiment, participants were randomly allocated to either an experimental or control condition and were administered experimental tasks. Allocation was not known to the research personnel until after analysis, whereupon data were unblinded. Both experiments received ethics approval from Deakin University: HEAG-H 24-2020 and HEAG-H 5-2022.

### 2.1. Sample

Experiment 1: Participants (N = 336; 261 women) were randomized and allocated to either control (n = 171) or experimental (n = 165) conditions. N = 164 completed the baseline demographics, (n = 88 control, n = 76 experimental), and N = 155 completed the immediate IMS follow-up outcome measures (n = 82 control, n = 73 experimental). N = 48 completed the Day 7 IMS (n = 28 control, n = 18 experimental), and N = 40 completed the Day 30 assessment (control n = 25, experimental n = 15). The sample age ranged from 18 to 88 years (M = 49.4, SD = 18.2). After randomization, participants provided demographic information and a measure of immediate mood state dysfunction by completing the Immediate Mood Scaler (IMS; Nahum et al. (2017)) prior to the experimental task. Participants repeated the IMS immediately after completing the experimental task. The experimental task was performed and outcome measurements were taken at baseline, seven days follow-up, and 30 days follow-up. Mood state was measured using the IMS immediately before and after the first administration, and again after the seven-day follow-up administration. Psychological distress scores were measured by the Depression, Anxiety, and Stress Scale (Lovibond & Lovibond, 1995) immediately before the first administration and after the seven- and thirty-day follow ups.

Experiment 2: A total of 247 participants provided affirmative consent, and 214 participants completed baseline assessments. A total of 184 participants were randomized, and 176 completed the follow-up assessment. Participants (N = 214; 152 women) comprised an international sample of English-speaking adults aged 18 to 69 years (M = 29.6, SD = 9.49). Participants were randomly allocated to one of four conditions: Happy–Angry (n = 52), Happy–Neutral (n = 44), Neutral–Happy (n = 38), or Control (n = 50). The demographic characteristics across each condition and experiment are outlined in Table 1 and Table 2.

Experiment 1 Power Analysis: Meta-analysis suggests that attentional biases tend to be smaller in size even when associated effects on mood are larger (Fodor et al., 2020), and so our power calculations suggested a minimum sample size of N = 193 to detect a small correlation of r = 0.2 at 80% power and 0.05 alpha.

Experiment 2 Power Analysis: A minimum sample of N = 200 (per condition, n = 50) was determined a priori using G*Power 3.1 software, assuming α = 0.05 with 80% power to detect a small effect size (Cohen’s f = 0.10).

### 2.2. Measures

#### 2.2.1. Psychological Distress

Depression, Anxiety, and Stress Scale: The Depression, Anxiety, and Stress Scale (DASS-21; Lovibond and Lovibond (1995)) is a 21-item screening tool for acute mental health risk, split equally across subscales of depressive, anxious, and stress symptoms. Each item is rated on a four-point scale (0 to 3), with higher scores representing greater severity. Scores higher than 4, 3, and 7 represent the presence of mild depressive, anxious, and stress symptoms, respectively. The three subscales of the DASS have good internal consistency: Depression α = 0.82; Anxiety α = 0.74; Stress α = 0.82 (Zanon et al., 2021). In this paper, we generally refer to symptomology measured by the DASS scales as psychological distress.

#### 2.2.2. Mood Dysfunction

Immediate Mood Scaler: The Immediate Mood Scaler (IMS; Nahum et al. (2017)) is a 22-item tool designed to capture momentary mood states. Participants rate their current mood state on a continuum using 7-point Likert scales (e.g., sad–happy, distracted–focused, sleepy–alert, fearful–fearless). The total score for this scale is the sum of the scores on all 22 items, and two subscales for depressive (seven items) and anxious (five items) mood states (Nahum et al., 2017). The score range for the total, depressive, and anxious mood state scales are 22 to 154, 6 to 42, and 5 to 37, respectively. Higher scores represent increasingly positive mood states. The internal consistency of the IMS is excellent for the total scale (α = 0.93) and for the subscales (α = 0.90 and 0.93 for depression and anxiety, respectively). In this paper, we generally refer to the scores on the IMS as mood dysfunction.

### 2.3. Experimental Task Description

The face-in-the-crowd task presents a participant with a 4-by-4 matrix of faces displaying a negative emotion and instructs them to seek out a randomly placed “target” face displaying a competing emotion. Participants view the face-image matrix and are instructed to find a target stimulus as fast as possible. In the first experiment, the target is a photo-realistic smiling face, and all other images are photo-realistic frowning faces (using an image package is commonly used in psychology research, e.g., Corriveau et al., 2024). In the control condition, the target is a five-petal flower (all other images are seven-petal flowers). In the second experiment, the target stimuli for the conditions included a single smiling face amongst an array of angry (i.e., threatening) faces (Happy–Angry condition); a single smiling face amongst neutral expressions (Happy–Neutral condition); and a single neutral expression amongst smiling faces (Neutral–Happy condition). The primary outcome is the time taken, or response latency, to find and click on the correct stimulus in milliseconds, and participants repeat the task 112 times in a single session, broken up into four blocks of 28 to allow for brief breaks. Overall, the task is generally completed in around five minutes. Differences in performance speed between different levels of group variables will be evaluated as potential attentional and cognitive biases. The use of face-in-the-crowd paradigms to detect attentional biases in clinical populations has been demonstrated (Rued et al., 2018); additional faces were generated using AI (https://Generated.photos; accessed 12 January 2022).

### 2.4. Presence of Attentional Bias

Consistent within the PST framework for addressing threat avoidance, we expected that increasing levels of psychological distress would correlate positively with latency; that is, greater levels of distress would increase fixation on threat stimuli and impede identification of positive or neutral stimuli. We contend that the presence of attentional biases would be observable through a correlation analysis of response latency with contemporaneous mood and psychological state, whereby we would observe distinct correlations between latency and these outcomes between the experimental conditions.

### 2.5. Analysis Plan

The efficacy of the face-in-the-crowd task in reducing mood state dysfunction was analyzed using three primary analyses.

Experiment 1: First, we examined changes in reported mood state scores using linear mixed models. Second, we examined the correlation between task response latency and contemporaneous mood scores. Finally, to assess whether AT might induce mood changes, we examined the scores of mood state dysfunction and psychological distress at each time point as predicted by earlier measurements, mediated by changes in AT task speed. Change scores are later-minus-earlier (i.e., T2-T1), so positive values indicate that a participant became slower over time.

Experiment 2 (AT): A one-way Analysis of Covariance (ANCOVA) was conducted to determine group differences in state anxiety, depression, and total psychological distress during the post-intervention assessment. The experimental condition was entered as the grouping variable, and post-intervention mood scores were dependent variables. Post hoc tests were assessed to examine simple between-group post-intervention differences.

## 3. Results

Experiment 1:

As shown in Table 1, the two groups were approximately equal for all outcome measurements at baseline. We observed a mild positive skew in each DASS subscale, which we corrected with square root transformations. The IMS scales were normally distributed. Finally, the mean response latency on the face-in-the-crowd task was highly negatively skewed and was therefore corrected by log10 transformations.

**Figure 1:**
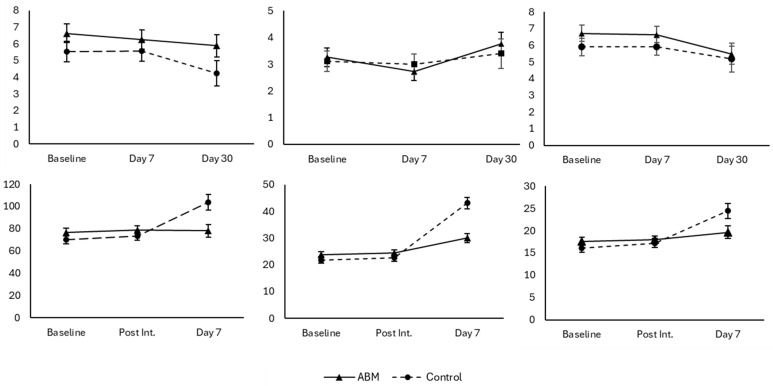
Experiment one outcomes. Left to right: DASS Depression, DASS Anxiety, DASS Stress, IMI Total, IMI Depression, and IMI anxiety.

Primary Outcomes:

Changes in Mood Over Time: As shown in Table 1 and Figure 1, we observed a significant time-by-group interaction for IMS Total, Depression, and Anxiety scores: F(2, 209) = 6.56, *p* = 0.002; F(2, 200) = 17.11, *p* < 0.001; F(2, 204) = 3.89, *p* = 0.021. For each outcome, the control group reported significant improvements over all three measurements, respectively: F(2, 209) = 11.39 B = 23.81, *p* < 0.001; F(2, 199) = 56.05, B = 15.05, *p* < 0.001; and F(2, 204) = 11.24, B = 5.86, *p* < 0.001. However, the only significant improvement observed in the experimental group was for IMS Anxiety: F (2, 203) = 6.81, B = 4.44, *p* = 0.001.

Changes in Psychological Distress: We observed significant main effects for time for each of the DASS Depression, Anxiety, and Stress symptoms: F (2, 189) = 4.36, *p* = 0.014; F(2, 181) = 4.99, *p* = 0.007; and F(2, 189) = 3.14, *p* = 0.045, respectively. Despite the lack of omnibus interaction effects, we observed different simple slopes between groups of DASS Anxiety and Stress. We observed significant quadratic slope effects for both treatment groups for Anxiety symptom scores (control: F [2, 184] = 9.04, B = 0.16, B2 = 0.24, *p* = 0.001; experimental F [2, 184] = 4.02, B = 0.22, B2 = 0.19, *p* =.019). Both groups reported an identical increase from day 7 to day 30 (MD = 0.40, t = 4.45, *p* < 0.001). However, the experimental group reported a significant decrease in Anxiety symptom scores from baseline to day 7 (MD = 0.18, t = 3.1, *p* = 0.003). We also observed significant decreases in Stress symptom scores for the experimental group: F(2, 194) = 3.09, B = −0.20, *p* = 0.034. Changes in the DASS scales were small for all outcomes and both groups (see Appendix A, Table A1).

Presence of Attentional Biases: We observed significant differences in the performance of each task according to log-latency at each time point; T1: Experimental Mean = 3.48, SD = 0.16; Control Mean = 3.62, SD = 0.17, t = −7.36, *p* = < 0.001, T2: Experimental Mean = 3.41, SD = 0.12; Control Mean = 3.46, SD = 0.12, t = −2.9, *p* = 0.004; and T3: Experimental Mean = 3.37, SD = 0.18; Control Mean = 3.38, SD = 0.09, t = −0.11, *p* = 0.92. However, we observed little evidence of attentional biases according to the correlation between mean response latency and mood state or psychological dysfunction; the only contemporaneous association between latency and any measure was observed in the experimental group for day 30 DASS Stress (r = 0.70, *p* = 0.007). Correlations are presented in Table 3.

Stability of Attentional Biases: The correlations between mood dysfunction, psychological distress, and changes in task latency were examined for each group to examine the stability of attentional biases (see Table 3). No associations were observed for the control group. In contrast, we observed small-to-moderate, positive correlations between baseline-to-day 7 latency changes and IMS Total, Depression, and Anxiety scores, as well as with DASS Depression. Further, comparably sized correlations were observed between baseline-to-day seven latency changes and IMS Depression and DASS Depression, as well as day 30 DASS Depression (see Table 3). In general, participants in the experimental group who improved at the face-in-the-crowd task reported better immediate mood states as recorded by the IMS, but worse past-week DASS depressive symptom scores.

**Figure 2:**
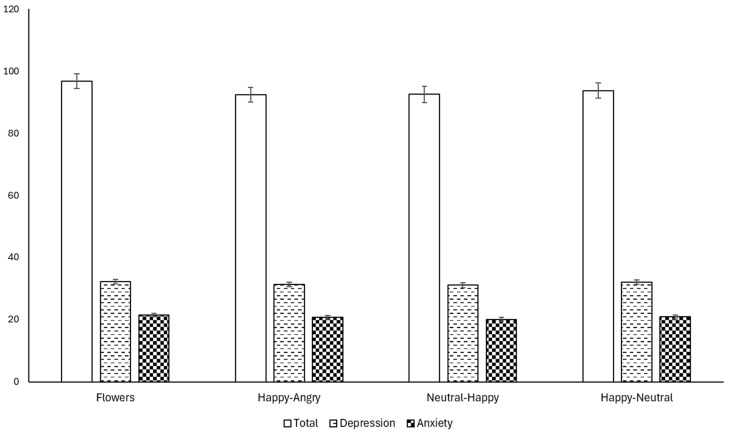
ANCOVA of each IMI index score post-intervention, adjusted for baseline values.

Experiment 2 (AMB) results:

We observed no significant between-groups differences post-intervention for IMS: total symptoms (overall model: F(4, 178) = 72.442, *p* < 0.001; condition: F(3, 178) = 0.713, *p* = 0.545; total symptoms T1; F(1, 178) = 272.973, *p* < 0.001), depression symptoms (overall model: F(4, 173) = 77.593, *p* < 0.001; condition: F(3, 173) = 0.462, *p* = 0.709; depression symptoms T1: F(1, 173) = 298.398, *p* = < 0.001), or anxiety symptoms (overall model: F(4, 173) = 72.433, *p* < 0.001, condition: F(3, 173) = 0.847, *p* = 0.47, anxiety symptoms T1: F(1, 173) = 280.41, *p* < 0.001) (see Table 2 and Figure 2).

## 4. Discussion

The current study aimed to investigate the effectiveness of a community sample face-in-the-crowd AT regarding mood states. Importantly, this investigation seeks to see if such biases and improvements can be observed/manipulated in a healthy sample, extending such findings beyond those seen in more clinical samples (e.g., Burns et al. (2025)). The results of the first experiment indicated that both the experimental and control tasks led to improvements in immediate mood states, but these improvements favored the control task. Additionally, while we observed reductions in psychological distress as measured by the DASS scales for the experimental condition and increases in DASS anxiety for both conditions, the effect sizes were small, raising questions about the practical significance of the task. Unexpectedly, we observed only a single indictor of attentional bias in the experimental conditions, with day seven latencies strongly associated with day thirty DASS stress. Contrary to expectations, we observed no change in the efficacy of the AT task across different emotional state targets.

Engaging in the attentional bias task positively influenced participant mood states, regardless of their assigned condition. However, we did not observe evidence of pre-existing attentional bias in our sample. Such bias only emerged after repeated exposure to the experimental task. Surprisingly, reductions in task performance were associated with increased positive mood states and decreased past-week depressive symptoms, contradicting prior research (Bourke et al., 2010). Task performance was related to outcome measurements, indicating mood effects associated with task improvements. Participants with higher pre- and post-task positive mood states exhibited decreased performance on day seven and improved psychological distress symptoms (DASS depression and stress scales) on day thirty (See Table 3). These small effects were not corroborated in our second experiment, which indicates that our findings might have been spurious.

If we are charitable, these findings are complex to interpret. While we expected improved task performance to be associated with immediate mood states and symptom reduction over time (as reported by Bourke et al. (2010)), we found that immediate mood improvement occurred after each administration of the control task. Neither task administration correlated with contemporaneous mood states or psychological distress scores (DASS). Our results align with experimental data suggesting initial mood improvements occur due to the transient cognitive control enhancement caused by directed training (Nejati et al., 2019). However, the lack of persistent training in our design might have limited the duration of the effect. One interpretation is that any initial attentional bias related to mood or psychological distress was likely absent in our sample, but the completion of all time points may have induced a protective bias.

Despite more promising findings in more recent meta-analyses (Matheson et al., 2019), the earlier research indicates an unreliable relationship between mood and attentional bias. Higher trait anxiety alone was often insufficient to induce detectable bias without additional stressors (e.g., including an initializing “stressor” task; Juth et al. (2005)). The emotional valence of the task also played a role in eliciting bias. Dodd et al. (2017) found that anxiety-related task performance deterioration occurred when emotional content was not emphasized. Our lack of observed difference between emotional states in the second experiment supports the speculation that attentional biases are not stable and might require either an acute increase in stress immediately prior to measurement, or some other factor that changes a participant’s expectations regarding the task. As our tasks did not aim to induce heightened anxiety and the control task lacked emotional references, our failure to detect initial bias might stem from the limitations of the experimental design rather than a complete rejection of attentional biases. However, our findings might also reflect the pattern of null findings across the previous AT literature in community samples (as opposed to clinical) (De Voogd et al., 2017; Waters et al., 2013). Further investigation into the role of attentional biases in sustaining or exacerbating distress states is needed, especially for the role of AT as a potential acute distress management tool.

It is also possible that our missing data is informative. In experiment one, we observed that our participants who dropped out of the study by day thirty reported significantly greater depression and worse mood states than those who completed all time points (DASS Depression: t(136) = 2.81, *p* = 0.005, MD = 2.19). Given that the level of psychological distress as measured by the DASS was universally low, this apparently paradoxical finding seems likely to be a spurious correlation. In contrast, the change in immediate mood state was far larger, as was the baseline difference between participants who dropped out and those who completed the experiment (t(94) = −7.42, *p* < 0.001, MD = 46.35). This large difference is predominantly driven by the experimental conditions; participants who dropped out before the day 30 time point consistently reported better mood states than those who completed the experiment.

This study had several limitations. In the first experiment, the small effect sizes in the experimental task compared to the control task cast doubt on its practical significance and clinical potential, especially considering the negative effect on psychological distress. The lack of correlation between task performance and changes in mood or distress implies an additional, unknown mechanism, contrasting with the successful use of the control task. As suggested earlier, it may be that an acute reduction in distress requires an acute state of stress, but more stable mood states are likely to have stable attentional characteristics that require sustained intervention. Relatedly, some studies suggest that completing simple, challenging tasks can temporarily reduce anxiety (Pine et al., 2020), which may explain the benefits of the control task. In a controlled VS task such as ours, the absence of emotional content or feedback might promote relaxation, leading to immediate but transient benefits without a durable distress reduction. However, our examination of this in our second experiment suggests that our optimistic findings from the first experiment might be type I errors. It is also possible that demographic factors were influential on our findings. Our sample ages differed between experiments, and there were minor age differences between participant groups despite randomization. Age is known to be influential in AT, since psychomotor speed tends to decrease and threat attendance biases towards negative stimuli as we age (Namaky et al., 2017). Further, both of our experimental samples were largely female, and while there is little evidence suggesting strong gender effects of AT in healthy adults (Pintzinger et al., 2016), this does suggest that further generalization may be limited.

Future research should explore more intensive intervention periods and repeated administrations within shorter intervals for improved mood effects. The initial inability to detect attentional bias may be due to insufficient distress in the sample, with subsequent exposures inducing the bias. This suggests heightened vigilance characteristics (Matheson et al., 2019; Moussally et al., 2016). Our findings raise questions about underlying mechanisms and advocate for further investigation into cognitive training for mood improvement. Finally, a major limitation of the study is the substantial attrition observed over time, with roughly 50% of participants being lost at each time point. Although there was no discernible demographic pattern of missingness, this raises concerns about the generalizability of the findings and the potential for selection bias. Our use of convenience sampling may also have been influential as the sample in experiment one was significantly older and reported lower levels of immediate distress compared to the second experiment. It is unclear what the impact of these differences might have on the findings, but it does appear inconsistent with our earlier assertion that greater levels of distress should be more receptive to AT in general.

## 5. Conclusions

Overall, our study finds little evidence to suggest that AT is effective for improving mood or psychological distress, at least insofar as attempting to visually stimulate emotional responses is concerned. Our most promising finding, that the control condition was particularly effective for improving mood states, suggests that the mechanisms of mood state changes through visual search paradigms might be unrelated to attention. However, our failure to corroborate this finding in the second experiment implies that the results are either spurious or reliant on confounding factors. We conclude that while attentional bias tasks might have some potential benefit as an intervention for mood states and dysfunction, the mechanisms underlying their benefits remain as elusive as reliable results.

## Figures and Tables

**Table 1:** Descriptive statistics for sample characteristics and outcome measures.

		Experimental		Control	
		*N*	%	*N*	%
***Age M (SD)***		47.7	(18.8)	51.7	(17.2)
***Gender***					
	Female	58	65.9%	49	64.5%
	Male	20	22.7%	16	21.1%
	Non-binary/Self-described	1	1.1%	0	0.0%
	Prefer not to say	9	10.2%	11	14.5%
***Educational Attainment***					
	Some secondary	5	5.7%	2	2.6%
	Completed secondary	8	9.1%	13	17.1%
	Vocational certificate	11	12.5%	13	17.1%
	Undergraduate degree	30	34.1%	21	27.6%
	Postgraduate degree	24	27.3%	14	18.4%
	Other	2	2.3%	3	3.9%
	Prefer not to say	8	9.1%	10	13.2%
***Employment***					
	Full-time	19	21.6%	17	22.4%
	Part-time	13	14.8%	14	18.5%
	Student	4	4.5%	2	2.6%
	Casual (non-contracted)	19	21.5%	13	17.1%
	Carer duties	3	3.4%	0	0.0%
	Not currently working	22	25.0%	19	25.0%
	Prefer not to say	8	9.1%	11	14.5%
***Outcome measures***		***M***	***SD***	***M***	***SD***
	IMS total baseline	76.88	25.09	69.75	28.91
	IMS total post-experiment	78.41	27.97	73.53	34.01
	IMS total day 7	76.32	45.34	98.72	51.90
	IMS depression baseline	23.59	9.34	21.74	10.23
	IMS depression post-experiment	24.32	10.08	22.66	11.60
	IMS depression day 7	29.18	12.29	40.39	7.00
	IMS anxiety baseline	17.49	7.63	16.01	8.05
	IMS anxiety post-experiment	17.74	7.80	17.26	8.91
	IMS anxiety day 7	18.75	9.88	23.61	9.60
	DASS depression baseline	6.52	5.40	5.36	4.85
	DASS depression day 7	6.14	5.41	5.64	5.49
	DASS depression day 30	5.24	4.03	3.33	3.56
	DASS anxiety baseline	3.26	3.07	2.96	3.22
	DASS anxiety day 7	2.69	2.85	3.04	3.79
	DASS anxiety day 30	3.57	2.64	2.55	1.37
	DASS stress baseline	6.68	4.39	5.73	4.62
	DASS stress day 7	6.52	4.63	5.96	4.74
	DASS stress day 30	4.96	3.18	4.77	2.55

**Table 2:** Experiment 2.

		Flowers		Happy-Angry		Happy-Neutral		Neutral-Happy	
		N	%	N	%	N	%	N	%
Age M (SD)		30.74	8.31	26.92	8.97	31.16	8.76	29.51	11.89
Gender									
	Female	36	72.0%	42	79.2%	29	63.0%	28	71.8%
	Male	14	28.0%	10	18.9%	11	23.9%	11	28.2%
	Non-binary	0	0.0%	1	1.9%	6	13.1%	0	0.0%
Educational Attainment									
	Some secondary	0	0.0%	2	3.8%	0	0.0%	0	0.0%
	Completed secondary	3	6.0%	6	11.3%	2	4.4%	3	7.7%
	Vocational certificate	6	12.0%	5	9.4%	3	6.5%	6	15.4%
	Bachelor’s degree	30	60.0%	33	62.3%	22	47.8%	22	56.4%
	Postgraduate degree	11	22.0%	7	13.2%	17	37.0%	7	17.9%
	Other	0	0.0%	0	0.0%	2	4.3%	1	2.6%
Employment									
	Full time	18	35.3%	10	18.9%	16	34.8%	14	35.9%
	Part time	10	19.6%	8	15.1%	9	19.6%	5	12.8%
	Casual	1	2.0%	2	3.8%	2	4.3%	1	2.6%
	Not currently employed	2	3.9%	3	5.7%	1	2.2%	1	2.6%
	Student	20	39.2%	30	56.6%	18	39.1%	18	46.2%
Outcome Measures		M	SD	M	SD	M	SD	M	SD
	IMS depression baseline	32.9	7.91	28.91	8.26	30.45	7.98	32.34	9.59
	IMS depression post-experiment	33.39	7.28	29.89	8.75	31.64	7.56	31.36	9.31
	IMS anxiety baseline	21.15	6.05	19.35	6.8	19.95	6.95	20	7.65
	IMS anxiety post-experiment	22	6.05	20.25	6.45	20.8	5.95	19.25	6.9
	IMS total baseline	99.5	20.54	88.51	23.24	91.47	23.49	93.54	30.74
	IMS total post-experiment	102	20.43	89.15	28.58	92.5	26.32	91.84	28.58

**Table 3:** Correlations between task latency, mood state, and psychological dysfunction.

	Control						Experimental					
	Log10 Latency			Latency Change			Log10 Latency			Latency Change		
	T1	T2	T3	T2-T1	T3-T1	T3-T2	T1	T2	T3	T2-T1	T3-T1	T3-T2
*T1*												
IMI Total	−0.189			0.123			−0.008			0.398 **		
IMS Depression	−0.193			0.146			0.030			0.481 ***		
IMS Anxiety	−0.116			0.061			−0.008			0.352 *		
DASS Stress	−0.028			0.189			−0.018			0.113		
DASS Anxiety	−0.009			0.203			−0.049			0.087		
DASS Depression	0.044			0.083			0.118			0.390 **		
*T2*												
IMS Total	−0.081	0.052		0.033	−0.500	0.500	0.090	0.119		0.242	0.110	0.280
IMS Depression	−0.098	0.020		0.001	−0.451	0.469	0.109	0.184		0.338 *	0.224	0.132
IMS Anxiety	−0.057	−0.039		0.062	−0.214	0.214	0.043	0.035		0.168	0.083	0.185
DASS Stress	−0.021	−0.048		0.213	−0.496	0.587	−0.133	−0.227		0.129	0.303	0.189
DASS Anxiety	0.006	−0.250		0.211	0.000	0.076	−0.101	−0.054		0.084	−0.108	0.173
DASS Depression	0.102	0.045		0.093	−0.674	0.281	0.055	0.238		0.332 *	0.128	0.483
*T3*												
IMS Total				0.155	0.288	−0.577				0.024	0.108	−0.024
IMS Depression				−0.121	0.073	−0.182				0.076	0.183	−0.238
IMS Anxiety				−0.116	−0.179	0.214				0.201	−0.018	−0.166
DASS Stress			−0.300	−0.091	−0.200	−0.300			0.703 **	0.188	−0.262	0.703
DASS Anxiety			0.258	−0.572	−0.775	0.258			0.325	0.085	−0.108	0.325
DASS Depression			0.131	−0.518	−0.393	0.131			0.234	0.485 *	0.003	0.234

Note. * *p* < 0.05, ** *p* < 0.01, *** *p* < 0.001.

## Data Availability

The data presented in this study are available on request from the corresponding author. The data are not publicly available due to institutional ethical restrictions.

## Abbreviations

The following abbreviations are used in this manuscript:

AT	Attention Training
DASS	Depression, Anxiety, and Stress Scale
IMS	Immediate Mood Scale
PST	Positive Search Training
VS	Visual Search

## Appendix A

**Table A1:** Paired *t*-tests for mood state and psychological dysfunction.

Control		T	df	*p*	SMD	
T1 DASS Stress	T2 DASS Stress	−0.93	72	0.354	−0.109	[−0.339, 0.121]
T1 DASS Stress	T3 DASS Stress	1.74	12	0.107	0.483	[−0.102, 1.051]
T2 DASS Stress	T3 DASS Stress	1.35	12	0.203	0.373	[−0.197, 0.93]
T1 DASS Anxiety	T2 DASS Anxiety	1.57	72	0.121	0.184	[−0.048, 0.415]
T1 DASS Anxiety	T3 DASS Anxiety	−1.39	10	0.193	−0.42	[−1.029, 0.207]
**T2 DASS Anxiety**	**T3 DASS Anxiety**	**−2.38**	**10**	**0.039**	**−0.717**	**[−1.37, −0.035]**
T1 DASS Depression	T2 DASS Depression	1.70	72	0.093	0.199	[−0.033, 0.43]
T1 DASS Depression	T3 DASS Depression	0.88	14	0.393	0.228	[−0.289, 0.737]
T2 DASS Depression	T3 DASS Depression	0.84	14	0.414	0.217	[−0.299, 0.726]
T1 IMS Total	T2 IMS Total	−1.30	71	0.200	−0.153	[−0.384, 0.08]
**T1 IMS Total**	**T3 IMS Total**	**−2.49**	**17**	**0.023**	**−0.587**	**[−1.081, −0.078]**
**T2 IMS Total**	**T3 IMS Total**	**−2.12**	**17**	**0.049**	**−0.499**	**[−0.983, −0.001]**
T1 IMS Depression	T2 IMS Depression	−0.90	71	0.374	−0.106	[−0.337, 0.127]
**T1 IMS Depression**	**T3 IMS Depression**	**−6.31**	**17**	**<0.001**	**−1.488**	**[−2.155, −0.8]**
**T2 IMS Depression**	**T3 IMS Depression**	**−5.22**	**17**	**<0.001**	**−1.231**	**[−1.839, −0.603]**
T1 IMS Anxiety	T2 IMS Anxiety	−1.34	71	0.183	−0.159	[−0.39, 0.075]
**T1 IMS Anxiety**	**T3 IMS Anxiety**	**−2.68**	**17**	**0.016**	**−0.632**	**[−1.132, −0.117]**
**T2 IMS Anxiety**	**T3 IMS Anxiety**	**−2.25**	**17**	**0.038**	**−0.531**	**[−1.02, −0.03]**
**Experimental**		**T**	**df**	***p***	**SMD**	**CI95%**
T1 DASS Stress	T2 DASS Stress	0.70	78	0.486	0.079	[−0.142, 0.299]
T1 DASS Stress	T3 DASS Stress	1.41	23	0.171	0.288	[−0.123, 0.694]
T2 DASS Stress	T3 DASS Stress	1.17	22	0.253	0.245	[−0.173, 0.657]
**T1 DASS Anxiety**	**T2 DASS Anxiety**	**3.10**	**78**	**0.003**	**0.348**	**[0.12, 0.575]**
T1 DASS Anxiety	T3 DASS Anxiety	−1.49	20	0.153	−0.324	[−0.76, 0.119]
**T2 DASS Anxiety**	**T3 DASS Anxiety**	**−2.75**	**18**	**0.013**	**−0.632**	**[−1.119, −0.131]**
T1 DASS Depression	T2 DASS Depression	1.99	78	0.051	0.224	[−0.001, 0.446]
T1 DASS Depression	T3 DASS Depression	0.45	23	0.656	0.092	[−0.31, 0.492]
T2 DASS Depression	T3 DASS Depression	1.02	22	0.319	0.213	[−0.203, 0.624]
T1 IMS Total	T2 IMS Total	−1.05	76	0.298	−0.119	[−0.343, 0.105]
T1 IMS Total	T3 IMS Total	−0.53	24	0.599	−0.107	[−0.499, 0.288]
T2 IMS Total	T3 IMS Total	0.19	24	0.852	0.038	[−0.355, 0.43]
T1 IMS Depression	T2 IMS Depression	−1.73	76	0.088	−0.197	[−0.422, 0.029]
**T1 IMS Depression**	**T3 IMS Depression**	**−2.25**	**24**	**0.034**	**−0.449**	**[−0.857, −0.033]**
**T2 IMS Depression**	**T3 IMS Depression**	**−2.16**	**24**	**0.041**	**−0.431**	**[−0.837, −0.017]**
T1 IMS Anxiety	T2 IMS Anxiety	−0.76	76	0.448	−0.087	[−0.31, 0.137]
T1 IMS Anxiety	T3 IMS Anxiety	−0.57	24	0.576	−0.114	[−0.506, 0.281]
T2 IMS Anxiety	T3 IMS Anxiety	−0.84	24	0.407	−0.169	[−0.562, 0.228]

Note. Hₐ μ Measure 1–Measure 2 ≠ 0.
